# The Incidence, Prevalence and Clinical Characteristics of Failure to Thrive in Children at King Abdullah Specialized Children Hospital, Riyadh, Saudi Arabia

**DOI:** 10.7759/cureus.51059

**Published:** 2023-12-25

**Authors:** Amal Alharbi, Mohammed Alanazi, Majid Alharbi, Abdulaziz Almonifi, Sultan Alshehri, Najd M AlNowaiser

**Affiliations:** 1 Pediatrics, King Abdulaziz Medical City Riyadh - Ministry of National Guard Health Affairs, Riyadh, SAU; 2 Internal Medicine, Prince Sultan Military Medical City, Riyadh, SAU; 3 College of Medicine, King Saud Bin Abdulaziz University for Health Sciences, Riyadh, SAU; 4 Neurology, King Abdulaziz Medical City Riyadh - Ministry of National Guard Health Affairs, Riyadh, SAU

**Keywords:** nutritional factors, weight faltering, failure to thrive, children, riyadh

## Abstract

Introduction

Failure to thrive (FTT) in children involves insufficient weight or height gain, affecting general and hospitalized populations which leads to cognitive and behavioral changes. Causes include inadequate caloric intake and underlying diseases (organic - OFTT) or psychosocial factors (non-organic - NOFTT). Our study in King Abdullah Specialized Children Hospital (KASCH) aims to assess FTT incidence, prevalence, and clinical characteristics, and also, to distinguish between different causes.

Methodology

It is a retrospective cohort study, conducted at KASCH, Riyadh, Saudi Arabia. This study includes children under three years old with documented FTT from 2017 to 2019. Data was collected from the hospital's electronic system and it was analyzed by the Statistical Package for the Social Sciences (IBM SPSS Statistics for Windows, IBM Corp., Version 29.0, Armonk, NY).

Results

Our study, including 214 FTT patients, revealed a balanced gender distribution of 109 males (50.9%), and 105 females (49.1%), with a majority of Saudi nationality 208 (97.2%). In most cases, 120 (56.1%) are in the 0-12 months age group. The prevalence of FTT was 26.75% (267 cases per 1000). Antenatal/post-natal features showed diverse delivery modes and NICU admissions. Chronic diseases like gastrointestinal diseases* *62 (29.1%), cardiac 50 (23.4%), and pulmonary 50 (23.4%) conditions were prevalent. Associations were found between NICU admission and pre-term births, birth weight status, and congenital anomalies, highlighting significant clinical correlations.

Conclusion

Our study concluded the significant burden of FTT at KASCH. Chronic diseases were playing a major role as a cause of FTT. Thus, emphasizing the causes and knowing the importance of addressing the prevalence and incidence is effective for appropriate intervention.

## Introduction

Failure to thrive (FTT) describes children with very low weight for age or height and those who do not maintain an appropriate growth pattern [1]. Within both the general and the hospitalized pediatric populations, FTT and malnutrition remain prevalent problems [2,3]. FTT is associated with adverse outcomes that include decreased cognition, learning difficulties, and behavior disturbances. Thus, the identification and management of FTT is crucial [4-6]. Inadequate caloric intake is the most common cause of FTT. Other causes include inadequate nutrient absorption, increased metabolic demands, or a combination of mentioned mechanisms [7].

Traditionally, risk factors for FTT are classified as non-organic or organic. Although multiple complex factors have a role in inadequate caloric intake, and, consequently, result in FTT. The prevalence of various cases can differ based on the patient’s age and specific underlying causes [1]. Organic failure to thrive (OFTT) is related to underlying diseases that play a role in inadequate caloric intake, which includes gastrointestinal (GIT) disease, cardiac disease, and some endocrine disorders [7]. In contrast, non-organic failure to thrive (NOFTT) includes psychosocial factors that affect caloric intake. For example, parents' educational level and socioeconomic status can have a significant impact on the child's nutritional status. Most cases of FTT are due to non-organic factors [1,8].

The prevalence of FTT depends on the population studied and the recognition criteria used. In the United States, in primary care clinics, up to 10% of attending children may have FTT, while in children who were admitted, the prevalence may reach up to 5% [2,9,10]. The rate of detection depends on the vigilance of individual physicians [6,9,10]. To our knowledge, no study had been done before to assess the incidence, prevalence, and clinical characteristics of FTT in Saudi Arabia, or the Middle East region. Thus, the aim of our study is to assess the incidence, prevalence, and clinical characteristics of FTT, and assess and compare the causes of OFTT among all overall cases of FTT.

## Materials and methods

This is a retrospective cohort, chart review study. This study was conducted at King Abdullah Specialized Children Hospital (KASCH), a tertiary care hospital in Riyadh, Saudi Arabia, for three years duration from January 1, 2017, to December 31, 2019. The study has IRB approval from King Abdullah International Medical Research Center (KAIMRC) with approval no.: NRC21R/424/10. The study included all children under three years who had documented a weight under the 5th percentile in the pediatric growth chart or drop 2 centiles from the last record in the growth chart, or had been diagnosed previously as a FTT. The characteristics of our population include male and female, aged from birth to three years, and nationality as Saudi or non-Saudi. Ethnicity and eligibility were not targeted. For exclusion, all children who did not fit the inclusion criteria.

The sample size was selected by a website known as RAOSOFT (http://www.raosoft.com/). Data was taken from the BESTCARE (https://bestcare.ezcaretech.com/) (health information system used to sort, organize, and monitor patient data inside the hospital), with the help of the KAIMRC to facilitate and approve the access. Data was obtained from the system by reviewing the records of patients who were diagnosed or at risk for FTT from January 2017 to December 2019. The entire population was targeted to be our sample. And so, our sampling technique was non-probability sampling, and the type was consecutive sampling type. We started in the recent year and moved backward. The total population was 800, only 214 fit our inclusion criteria.

Data was entered in an Excel sheet (MS Excel for Office 365 MSO, Version 16.0, Microsoft® Corp., Redmond, WA). The research team members were assigned to review the data. The datasheet file was kept anonymous with no names or identities only coded numbers were used. A comprehensive statistical analysis was conducted on the dataset, encompassing both descriptive and inferential methodologies. Firstly, a descriptive is conducted to summarize the demographic characteristics of the patients, which includes age, gender, and prevalence classification. And then presented these data as a percentage and numbers. This provides an overview of the study population, prevalence, and incidence. Fisher’s Exact test/Chi-Square tests are used to find an association between NICU admission and other features. Statistical significance is established at a p-value of 0.05 or lower and a 95% confidence interval. All statistical analyses are executed using the Statistical Package for the Social Sciences (IBM SPSS Statistics for Windows, IBM Corp., Version 29.0, Armonk, NY).

## Results

Our study includes 214 patients, and the sociodemographic characteristics revealed a balanced gender distribution (50.9% male, 49.1% female). The majority were Saudi nationals (97.2%). The age distribution ranged from 0 to 36 months, commonly the 0-12 months age group (56.1%) with a mean age of 13.1 months. Weight averaged at 6.41 kg, and height at 67.4 cm (Table 1).

**Table 1:** Sociodemographic characteristics of patients (n=214)

Characteristics		Frequency n (%)
Gender	Male	109 (50.9)
	Female	105 (49.1)
Age (months)	0-12	120 (56.1)
	13-24	57 (26.6)
	25-36	37 (17.3)
	Mean (±SD)	13.1 (±9.6)
	Range	0-36
Nationality	Saudi	208 (97.2)
	Non-Saudi	6 (2.8)
Weight (Kg)	Mean (±SD)	6.41 (±6.0)
	Range	0.8-14.9
Height (cm)	Mean (±SD)	67.4 (±13.2)
	Range	33.5-103

Table 2 shows that in a population of 800 children who were at risk for FTT and visited KASCH between January 1, 2017, and December 31, 2019; only 214 patients fit the inclusion criteria and were diagnosed as FTT. The prevalence of FTT was calculated as 26.75%. The annual incidence rates showed 100 cases per 1000 people in 2017, and 82.5 cases per 1000 people in 2018, while in 2019, it was 85 cases per 1000.

**Table 2:** Prevalence and incidence rate of failure to thrive (FTT) of children Total population (n=800)

Prevalence of FTT		
Frequency of cases Prevalence		
Prevalence From Jan 2017 to Dec 2019	214 FTT Cases	26.75%
Incidence of FTT		
Frequency of Cases Incidence rate		
Incidence of FTT in 2017	80 FTT Cases	100/1000 People
Incidence of FTT in 2018	66 FTT Cases	82.5/1000 People
Incidence of FTT in 2019	68 FTT Cases	85/1000 People

Table 3 shows that the distribution of antenatal and post-natal features revealed that 137 (64%) were born at term, 56 (26.2%) pre-term, and 21 (9.8%) had missing data. Normal spontaneous vaginal delivery (NSVD) occurred in 110 (51.4%), assisted delivery in two (0.9%), and cesarean section (CS) in 72 (33.6%). About 74 (34.6%) had a normal birth weight, 91 (42.5%) experienced NICU admission, and 75 (35%) had congenital anomalies.

**Table 3:** Different antenatal and post-natal features of children (n=214) n: Frequency, %: percentage, NSVD: normal spontaneous vaginal delivery, CS: cesarean section

Variable		Frequency n (%)
Term	Term	137 (64)
	Pre-term	56 (26.2)
	Unknown	21 (9.8)
Mode of Delivery	NSVD	110 (51.4)
	Assisted	2 (0.9)
	CS	72 (33.6)
	Unknown	30 (14.0)
Normal Birth Weight	Yes	74 (34.6)
	No	70 (32.7)
	Unknown	70 (32.7)
NICU Admission	Yes	91 (42.5)
	No	102 (47.7)
	Unknown	21 (9.8)
Congenital Anomalies	Yes	75 (35.0)
	No	139 (65.6)

**Figure 1:**
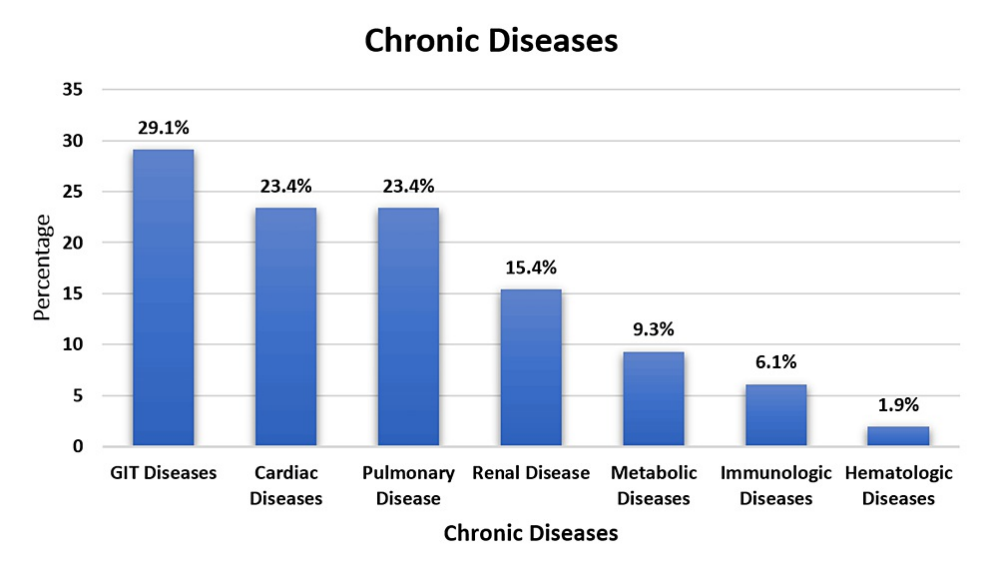
Different chronic diseases in FTT children (n=214) FTT: failure to thrive, GIT: gastrointestinal

Figure 1 shows different chronic diseases in FTT; some children have multiple diseases. GIT diseases are the most prevalent at 62 (29%), followed closely by cardiac and pulmonary diseases; both had 50 (23.4%). Renal diseases account for 33 (15.4%), metabolic diseases for 20 (9.3%), immunologic diseases for 13(6.1%), and hematologic diseases for 4 (1.9%).

Table 4 shows the laboratory profile of FTT children. Metabolic screening was conducted in 20 (9.3%), with 126 (58.9%) not screened, and 68 (31.8%) unknown. Anemia was found in 68 (31.8%), and the mean hemoglobin level was 11.6 g/dl. Mean corpuscular volume (MCV) in femtoliters (fL) shows a mean of 82.6 (±9.5) with a range from 57.7 to 120. Additionally, the mean corpuscular hemoglobin (MCH) in picograms (pg) has a mean of 26.9 (±3.4) with a range spanning from 15.6 to 38.4. Celiac screening was performed in 49 (22.9%), with tissue transglutaminase IgA mean at 10.4 units/L. Thyroid screening was done in 82 (38.3%), with a mean thyroid-stimulating hormone (TSH) level of 2.8 mU/L and free T4 at 13.2 ug/dL.

**Table 4:** Laboratory profile of FTT children (n=214) FTT: failure to thrive, MCV: mean corpuscular volume, MCH: mean corpuscular hemoglobin, TSH: thyroid-stimulating hormone

Test		Frequency n (%)
Metabolic Screening	Yes	20 (9.3)
	No	126 (58.9)
	Unknown	68 (31.8)
Anemia	Yes	68 (31.8)
	No	130 (60.7)
	Unknown	16 (7.5)
Hemoglobin Level (g/dl)	Mean (SD)	11.6 (±1.7)
	Range	6.1-21.8
MCV (fL)	Mean (SD)	82.6 (±9.5)
	Range	57.7-120
MCH (pg)	Mean (SD)	26.9 (±3.4)
	Range	15.6-38.4
Celiac Screening	Yes	49 (22.9)
	No	27 (12.6)
	Unknown	138 (64.5)
Tissue Transglutaminase IgA (units/L)	Mean (SD)	10.4 (±27.3)
	Range	0.0-218.6
IgA Level	Mean (SD)	1.7 (±5.0)
	Range	0.1-42.0
Thyroid Screening	Yes	82 (38.3)
	No	39 (18.2)
	Unknown	93 (43.5)
TSH Level (mU/L)	Mean (SD)	2.8 (±2.4)
	Range	0.33-15.1
Free T4 (ug/dL)	Mean (SD)	13.2 (±6.6)
	Range	2.34-72.0

Table 5 shows the association between NICU admission and various features in FTT children. Notable findings reveal a significant correlation between NICU admission and pre-term births (78.6%) (p < 0.001), normal birth weight status (29.7%) (p < 0.001), and the presence of congenital anomalies (70.7%) (p < 0.001). Moreover, age, gender, and mode of delivery do not show significant associations with NICU admission.

**Table 5:** Association of NICU admission with different features in FTT children FTT: failure to thrive, a: Fisher’s Exact test, b: Chi-Square test, n: Frequency, %: percentage, NSVD: normal spontaneous vaginal delivery, CS: cesarean section

Variable		NICU Admission		Sig. Value
		No n (%)	Yes n (%)	
Age (Month)	0-12	66 (55.0)	54 (45.0)	0.588^ b^
	13-24	36 (63.2)	21 (36.8)	
	25-36	21 (56.8)	16 (43.2)	
Gender	Male	67 (61.5)	42 (38.5)	0.229^ b^
	Female	56 (53.5)	49 (46.7)	
Nationality	Saudi	121 (58.2)	87 (41.8)	0.405^a^
	Non-Saudi	2 (33.3)	4 (66.7)	
Term	Term	93 (67.9)	44 (32.1)	<0.001^ b^
	Pre-term	12 (21.4)	44 (78.6)	
Mode of Delivery	NSVD	66 (60.0)	44 (40.0)	0.110^a^
	Assisted	1 (50.0)	1 (50.0)	
	CS	32 (44.4)	40 (55.6)	
Normal Birth Weight	Yes	52 (70.3)	22 (29.7)	<0.001^ b^
	No	25 (35.7)	45 (64.3)	
	Unknown	46 (65.7)	24 (34.3)	
Congenital Anomalies	Yes	22 (29.3)	53 (70.7)	<0.001^ b^
	No	98 (72.1)	38 (27.9)	
Anemia	Yes	42 (61.8)	26 (38.2)	0.203^ b^
	No	68 (52.3)	62 (47.7)	
Celiac Screening	Yes	31 (63.3)	18 (36.7)	0.767^ b^
	No	18 (66.7)	9 (33.3)	
Thyroid Screening	Yes	47 (57.3)	35 (42.7)	0.157^b^
	No	17 (43.6)	22 (56.4)	

Table 6 shows the association between NICU admission and various chronic conditions in FTT children. Significant associations were found between NICU admission and cardiac diseases (70%) (p<0.001), pulmonary diseases (60.0%) (p=0.005), and GIT diseases (59.7%) (p<0.001). Children with these conditions had a higher likelihood of NICU admission. Conversely, renal diseases, immunologic diseases, metabolic diseases, and hematologic diseases did not show statistically significant associations with NICU admission.

**Table 6:** Association of NICU admission with different chronic conditions in FTT children FTT: failure to thrive, GIT: gastrointestinal, a: Fisher’s Exact test, b: Chi-Square test

Disease		NICU Admission		Sig. Value
		No n(%)	Yes n(%)	
Cardiac Diseases	Yes	15 (30)	35 (70)	<*0.001*^ b^
	No	106 (65.4)	56 (34.6)	
Renal Diseases	Yes	15 (45.5)	18 (54.5)	0.142^ b^
	No	106 (59.2)	73 (40.8)	
Pulmonary Diseases	Yes	20 (40.0)	30 (60.0)	*0.005*^ b^
	No	101 (62.3)	61 (37.7)	
GIT Diseases	Yes	25 (40.3)	37 (59.7)	<*0.001*^ b^
	No	96 (64.0)	54 (36.0)	
Immunologic Diseases	Yes	7 (53.8)	6 (46.2)	0.808^ b^
	No	114 (57.3)	85 (42.7)	
Metabolic Diseases	Yes	9 (45.0)	11 (55.0)	0.222^ b^
	No	75 (59.5)	51 (40.5)	
Hematologic Diseases	Yes	3 (75.0)	1 (25.0)	0.637^a^
	No	118 (56.7)	90 (43.3)	

## Discussion

FTT is inadequate weight gain in children. Definitions are described by using the growth chart, either a weight less than the 5th percentile, or a drop in weight for more than or equal to 2 percentile lines [11]. Causes include inadequate caloric intake, nutrient absorption issues, and increased metabolic demands [12]. FTT is classified as organic (linked to diseases) or non-organic (psychosocial factors). Most cases are non-organic, influenced by factors like parental education and socioeconomic status. Our findings provide valuable insights into various aspects of FTT, from sociodemographic characteristics to chronic conditions and associated features. Our aim was to assess the incidence and prevalence and to compare the causes of OFTT. In this discussion section, we will interpret these results in the context of existing literature, drawing connections and highlighting novel contributions to the understanding of FTT.

The balanced gender distribution observed in our study shows that there is relatively equal prevalence of FTT among male and female infants. This is in contrast with the previous study by Vaghari et al. (2002), which shows that malnutrition and FTT were more in boys than girls [13], while Habibzadeh et al. (2015) show that the prevalence of growth failure in female infants was somewhat more than that in the males [14]. The majority being Saudi nationals (97.2%) may reflect the demographic composition of the local population.

Moreover, the high prevalence of FTT, with 267 cases per 1000 people or 26.7%, indicates a significant burden within the studied population. While, a study by Ross et al. (2017) shows that the community prevalence of FTT in high-income countries is reported to be 1-10% under two years of age, with the United States seeing 5-10% of children presenting in primary care settings and 3-5% of children in hospital settings [15]. For the incidence rates from 2017 to 2019 (100 to 85 cases per 1000 people), a slight reduction in the number of cases will make us ask a question if there are any causes that changed and reduced the cases. However, caution is needed in attributing these changes solely to healthcare interventions, as external factors like socioeconomic conditions can also influence FTT rates.

The distribution of antenatal and post-natal features, such as weight birth and prevalence of NICU admission, aligns with a previous study by Moreno et al. (2023) and shows the readmission among neonates with lower birth weight increases as gestational age increases [16]. The higher prevalence of NICU admission in pre-term births (78.6%) is consistent with the known vulnerability of premature infants to health complications. Another study highlights that the multivariate analysis of the data indicated that preterm delivery is independently associated with a greater prevalence of NICU admission among infants with very low birth weight (VLBW) [17]. The association between NICU admission and congenital anomalies (70.7%) is a critical finding, emphasizing the need for specialized care for this subgroup of FTT children. Chung et al. (2020) show that the congenital anomaly increased the risk of in-hospital admission and mortality and was associated with short-term neonatal morbidities in VLBW infants [18].

The prevalence of chronic diseases in FTT children corresponds with existing knowledge. GIT diseases, cardiac diseases, and pulmonary diseases being the most prevalent are consistent with literature highlighting the multifactorial etiology of FTT, often involving GIT and respiratory issues. Rodríguez et al. (2011) show the associations between malnutrition or FTT and the incidences of GIT or respiratory infections in children under five years of age [19]. The laboratory profile further complements these findings, with anemia being a common feature, aligning with studies emphasizing the importance of hematological assessments in FTT diagnosis. Similarly, previous studies show that malnutrition and anemia show a significant association in preschoolers, with both conditions affecting about half of the study group [20,21].

Our study also established significant associations between NICU admission and specific features. The strong correlation with pre-term births, normal birth weight, and the presence of congenital anomalies reinforces the understanding that certain subgroups of FTT children are at a higher risk of requiring intensive care. In study by Peterson et al. (2023) shows that there was a link in patient characteristics between prior stay in the NICU and having a low birth weight, and the vulnerability of pre-term infants and those with congenital anomalies to multiple comorbidities [22].

Similarly, the association between NICU admission and chronic conditions reveals significant correlations with cardiac, pulmonary, and GIT diseases. Yilmaz et al. (2022) show the impact and association of malnutrition and FTT on mortality, postoperative infection, and length of hospitalization in children undergoing surgery for congenital heart disease [23]. These findings underscore the importance of considering underlying chronic health conditions in the management and care planning for FTT children. The lack of significant associations with renal, immunologic, metabolic, and hematologic diseases may suggest a more complex interplay of factors influencing NICU admission in these cases.

Study limitations

While the study offers valuable insights, certain limitations should be acknowledged. The retrospective nature of the study and the reliance on medical records introduce the possibility of incomplete or inaccurate data. Additionally, the study's focus on a specific demographic may limit the generalizability of findings to other populations.

## Conclusions

Our study provides a nuanced understanding of FTT, encompassing sociodemographic characteristics, prevalence, chronic conditions, and associated features. The findings contribute to the existing literature by shedding light on specific subgroups of FTT children at higher risk of NICU admission and emphasizing the importance of considering underlying chronic health conditions in clinical management. The study sets the stage for further research, guiding interventions aimed at improving the outcomes of FTT children.
